# Gene Regulatory Networks for Enhanced Vision-Based Robot Control: A Bio-Inspired Approach

**DOI:** 10.3390/s26061742

**Published:** 2026-03-10

**Authors:** Chourouk Guettas, Foudil Cherif, Ammar Muthanna, Mohammad Hammoudeh, Abdelkader Laouid

**Affiliations:** 1 LESIA Laboratory, University of Biskra, P.O. Box 145 RP, Biskra 07000, Algeria; guettas-chourouk@univ-eloued.dz (C.G.); cherif.foudil@univ-biskra.dz (F.C.); 2 Institute of Computer Science and Telecommunications, RUDN University, 117198 Moscow, Russia; mutkhanna-as@rudn.ru; 3 Department of Information and Computer Science, King Fahd University of Petroleum & Minerals, Dhahran 31261, Saudi Arabia; mohammad.hammoudeh@kfupm.edu.sa; 4 LIAP Laboratory, El Oued University, P.O. Box 789, El Oued 39000, Algeria

**Keywords:** gene regulatory networks, vision-based robotics, bio-inspired control, robot learning, evolutionary robotics

## Abstract

Vision-based robot control remains a significant challenge due to the sample inefficiency and prolonged training times associated with traditional deep reinforcement learning methods. We propose a novel approach inspired by biological gene regulation, leveraging Gene Regulatory Networks (GRNs) for efficient and robust robot control. In our approach, robot states are encoded as gene expression levels, and evolutionary optimization is used to learn GRN parameters that map raw visual inputs to motor commands. We evaluate this method on the KukaDiverseObjectEnv benchmark, where robots must grasp diverse objects using only RGB images. Our GRN-based controller achieves a 57.5% success rate while reducing training time by 13.7× compared to Proximal Policy Optimization baselines. It also outperforms NEAT, standard reinforcement learning algorithms, and deep Q-learning in terms of both efficiency and performance. The controller maintains 91.8% performance under noisy visual conditions. This bio-inspired design naturally enables hierarchical control via expression cascades, computational efficiency through bounded dynamics, and temporal reasoning without explicit memory modules.

## 1. Introduction

Vision-based robot control is a challenging problem in robotics, requiring systems to process high-dimensional visual inputs and generate appropriate motor commands in real time. Traditional approaches relied heavily on deep reinforcement learning methods, which demonstrated remarkable success across various robotic applications [1]. However, these methods often suffer from sample inefficiency, requiring extensive training periods and substantial computational resources [2]. Recent studies, e.g., [3], highlight fundamental difficulties in real-world deployment due to the complexity and cost of physical world interactions.

Bio-inspired approaches provide alternatives to traditional neural network-based methods. Evolutionary robotics explores various biological metaphors for robot control, including neural evolution techniques that simultaneously optimize network topology and parameters [4,5]. However, most existing bio-inspired methods focus on neural architectures rather than exploring other sophisticated biological control mechanisms.

This article introduces an application of Gene Regulatory Networks (GRNs) to vision-based robot control. GRNs orchestrate complex cellular behaviors in biological systems through dynamic gene expression patterns [6]. These networks exhibit hierarchical organization, temporal dynamics without explicit memory mechanisms, robustness through distributed decision-making, and computational efficiency via sparse connectivity [7,8]. Despite extensive computational biology research, applying GRN principles to robotic control remains unexplored.

Our approach represents robot states as gene expression levels and employs evolutionary optimization to learn regulatory network parameters that map visual inputs to motor commands. Drawing inspiration from biological regulatory mechanisms, we develop controllers that exhibit robustness, adaptability, and efficient resource utilization.

The main contributions of this article are (1) a novel GRN-based control architecture for vision-based robotics that addresses sample inefficiency and training time limitations, (2) an evolutionary optimization specifically adapted for GRN parameters with biologically motivated constraints, (3) a comprehensive evaluation on the KukaDiverseObjectEnv benchmark showing a 57.5% success rate with 13.7× faster training compared to established baselines, and (4) a systematic ablation analysis validating that performance improvements stem from fundamental biological principles rather than superficial biomimicry.

The remainder of this article is organized as follows: Section 2 provides an overview of Gene Regulatory Networks and their computational properties. Section 3 reviews vision-based robot control methods and positions our approach within the existing literature. Section 4 presents the mathematical formulation of our GRN model, system architecture, and evolutionary optimization framework. Section 5 describes the experimental setup, presents results, and discusses performance analysis, robustness evaluation, and ablation studies. Section 6 concludes and outlines future work directions.

## 2. GRNs Overview

GRNs govern cellular behavior through intricate gene–regulatory protein interactions. Unlike simple input–output mappings, genes regulate each other’s expression through complex feedback loops, creating control systems where gene expression levels serve as distributed state variables. This biological architecture evolved over millions of years to produce remarkably robust and adaptive control mechanisms that maintain cellular function despite environmental perturbations and molecular noise [7].

The mathematical foundation of GRNs relies on differential equations describing gene expression dynamics. In biological systems, transcription factors bind to gene promoter regions with cooperative binding kinetics, creating sigmoidal dose–response relationships. This cooperative binding phenomenon follows Hill kinetics, described by the following equation:

$$\mathrm{Response}=\frac{{\left[\mathrm{Input}\right]}^{n}}{K^{n}+{\left[\mathrm{Input}\right]}^{n}}$$

where *n* represents the cooperativity coefficient, and *K* is the dissociation constant [9]. This equation naturally implements threshold-based switching behavior without explicit programming, a property that proves invaluable for control applications.

Critical to biological GRNs are three key properties that distinguish them from artificial neural networks. First, bounded dynamics ensure gene expression remains within finite ranges, providing inherent stability [10]. Second, sparse connectivity patterns (typically 15–25% of possible gene–gene interactions that are active) emerge from evolutionary optimization, reducing computational complexity while maintaining rich dynamics [11]. Third, intrinsic noise tolerance allows molecular fluctuations to be filtered rather than amplified, creating naturally robust control systems [12]. Computational applications of GRNs have demonstrated success in synthetic biology, where researchers have engineered artificial gene circuits to implement logic functions, oscillators, and bistable switches in living cells [13]. These engineered biological systems prove that GRN principles can be deliberately designed to achieve specific input–output behaviors, validating their computational utility beyond natural biological contexts.

The adaptation of GRNs to robotics addresses fundamental limitations that have long plagued existing approaches. Traditional reinforcement learning algorithms require extensive exploration phases and suffer from sample inefficiency, often needing millions of interactions to achieve competent performance [1]. Neural evolution approaches, while avoiding the challenges of gradient-based optimization, lack the structured constraints that guide biological systems toward stable and robust solutions [4]. Deep learning methods operate in unbounded parameter spaces that complicate optimization and limit interpretability, which is critical in robotic applications [14].

GRNs offer a fundamentally different paradigm by providing structured search spaces defined by biological constraints. The bounded nature of gene expression prevents the parameter explosion that can destabilize neural network training, while sparse connectivity reduces the effective dimensionality of the optimization problem without sacrificing representational power [8]. Most importantly, temporal dynamics emerge naturally from gene regulation and decay mechanisms, eliminating the need for explicit memory architectures or recurrent connections that complicate traditional neural approaches [15]. These properties collectively enable efficient evolutionary optimization while maintaining the representational power necessary for complex control tasks. By leveraging millions of years of biological evolution, we can access control strategies that have already been tested and refined by natural selection, offering a principled foundation for developing robust robotic controllers.

## 3. Related Work

We organize the relevant literature into distinct categories to better contextualize our GRN-based approach within the broader landscape of robotic grasping and bio-inspired control methods.

### 3.1. Vision-Based Robotic Grasping

Robot grasping evolved from analytical approaches requiring exact object models to data-driven methods that learn from experience. Early systems utilized hand-engineered features and geometric primitives [16,17], but these strategies failed drastically when faced with novel objects or cluttered scenes due to their reliance on complete prior knowledge of object physics and geometry [18].

#### 3.1.1. Deep Learning Approaches

This limitation triggered the transition to deep learning methodologies, where CNN implementation recognized grasp configurations directly from RGB-D images [19,20]. Lenz et al. [20] demonstrated that neural networks could learn graspable areas without explicit feature engineering, achieving considerable improvement over classical methods. Nevertheless, these initial deep learning systems required large, labeled datasets and struggled with generalization to diverse object categories.

The advent of deep reinforcement learning solved the data labeling bottleneck through autonomous policy learning via trial-and-error interaction [21,22,23]. QT-Opt [24] exemplified this paradigm, achieving 96% grasp success rates on novel objects via distributed training on seven robots for 800 h. While impressive, this result highlighted the substantial computational resources required. To enhance sample efficiency, researchers have employed off-policy techniques that reuse previously collected data, making training significantly faster than on-policy alternatives that necessitate new data for each update.

#### 3.1.2. Multi-Modal and Multi-Action Systems

Following single-primitive limitations, multi-action approaches were developed to tackle complex scenarios. Zeng et al. [25] demonstrated that integrating pushing and grasping actions achieved 13% higher success rates in cluttered settings compared to grasp-only methods. That research confirmed that pre-manipulation actions could enable more stable grasping results, motivating further studies on object shifting and rearrangement methods [18].

The combination of multiple sensory modalities enhances system robustness. Visual–tactile fusion methods merge global visual information with local tactile feedback for contact estimation and slip detection [26,27,28]. Such systems demonstrate advantages in occluded situations where vision alone does not convey enough information for stable grasping decisions.

#### 3.1.3. Generative and Foundation Model Approaches

Recent advances have focused on generative methods for grasp synthesis. Zhang et al.’s GraspXL [29] synthesizes varied grasp motions at scale with transformer architectures, while their ArtiGrasp work [30] extends this to bi-manual manipulation with articulated objects. Concurrently, diffusion models have been applied to dexterous grasping via DexDiffuser [31], generating 24-DOF hand configurations for intricate objects. These generative approaches mark a departure from discriminative grasp detection towards holistic motion synthesis.

### 3.2. Vision–Language Integration in Robotics

Language-guided manipulation has become a prominent direction, with frameworks such as “Grasp as you say” [32] enabling natural language definition of grasping actions. This research connects high-level semantic comprehension with low-level motor control, although current implementations are still constrained to preset vocabulary and basic spatial relations.

Vision–language models represent a significant advancement in creating generalist agents that leverage large-scale pretraining for semantic understanding [32]. These systems enable multi-task manipulation, zero-shot transfer, and semantic reasoning by interpreting natural language commands, though they typically require substantial computational infrastructure and extensive pretraining datasets.

### 3.3. Bio-Inspired Approaches in Robotics

Bio-inspired methodologies provide alternative approaches to the robustness issues faced by traditional learning methods. Although evolutionary algorithms were proven effective for parameter optimization [5,33], applying biological regulatory mechanisms to manipulation control has received limited attention.

#### 3.3.1. Evolutionary and Neural Evolution Methods

Evolutionary robotics explores various biological metaphors for robot control, including neural evolution techniques that simultaneously optimize network topology and parameters [4,5]. However, most existing bio-inspired methods focus on neural architectures rather than exploring other sophisticated biological control mechanisms.

#### 3.3.2. Central Pattern Generators and Neurodynamics

Neurodynamics-inspired techniques such as central pattern generators (CPGs) [34,35,36] show promise for real-time collision-free planning without requiring global knowledge, indicating that biological principles may overcome current limitations in adaptability and environmental robustness. These approaches demonstrate that biological control mechanisms can provide natural rhythmic behaviors and adaptive responses.

#### 3.3.3. Gene Regulatory Networks in Computational Applications

Despite extensive computational biology research, applying GRN principles to robotic control remains unexplored. Computational applications of GRNs have demonstrated success in synthetic biology, where researchers have engineered artificial gene circuits to implement logic functions, oscillators, and bistable switches in living cells [13]. These engineered biological systems prove that GRN principles can be deliberately designed to achieve specific input–output behaviors, validating their computational utility beyond natural biological contexts.

GRNs offer fundamentally different paradigms by providing structured search spaces defined by biological constraints. The bounded nature of gene expression prevents parameter explosion, while sparse connectivity reduces optimization complexity without sacrificing representational power [8]. Temporal dynamics emerge naturally from gene regulation and decay mechanisms, eliminating the need for explicit memory architectures [15].

### 3.4. Current Limitations and Research Gap

Notwithstanding these advancements, significant challenges remain. Presently, vision-based systems exhibit variable performance across different object categories, with success rates fluctuating between 60% and 95%, contingent upon object attributes and environmental conditions [37]. Furthermore, most methodologies display limited robustness to hardware modifications, necessitating considerable retraining whenever gripper configurations or camera placements are altered [22,38].

Table 1 compares the major learning-based robotic grasping methodologies, emphasizing their fundamental contributions, common applications, and major advantages. The comparison illustrates how research has progressed from hand-engineered features to complex learning paradigms, while also showing the missing link that bio-inspired methods seek to fill.

Our research presents GRNs as a bio-inspired control framework for vision-based manipulation, leveraging the stability and adaptability properties inherent to biological regulatory systems [39] to address the robustness and efficiency issues that current learning-based methods have not been able to resolve. This represents the first application of evolved GRNs to vision-based robotic grasping, combining biological regulatory principles with evolutionary optimization for efficient robot control.

## 4. The Proposed Bio-Inspired Approach

We illustrate in this section how GRNs are applied to vision-based robot control, giving the mathematical formulation, system architecture, and optimization framework. We begin by describing the core dynamics that govern gene expression, including the mapping from sensory inputs to regulatory signals and the generation of motor commands from the evolved gene expression patterns.

### 4.1. Mathematical Formulation

The proposed GRN model consists of n=50 genes, where each gene *i* has an expression level $x_{i}\left(t\right)\in \left[0.0,1.0\right]$ at a discrete time step *t*. The choice of 50 genes provides sufficient complexity for control tasks while maintaining computational tractability. Equation (1) represents the core dynamics, which are modeled using discrete-time updates inspired by differential equation dynamics.

(1) $$x_{i}\left(t+1\right)=x_{i}\left(t\right)+\Delta t\cdot \left[f_{i}\left(s,x\right)-\delta_{i}x_{i}\left(t\right)+\eta_{i}\left(t\right)\right]$$

Equation (1) describes the discrete-time gene expression dynamics, where the gene expression vector $x={\left[x_{1},x_{2},\ldots ,x_{n}\right]}^{T}$ captures the current state of the regulatory network. The sensory input vector $s\in R^{8}$ combines visual and proprioceptive information, where the first four dimensions represent processed visual features and the remaining four dimensions encode proprioceptive feedback from robot joint states. The activation function $f_{i}\left(s,x\right)$ determines how gene *i* responds to environmental stimuli and regulatory influences from other genes. Each gene has an associated decay rate $\delta_{i}\in \left[0.05,1.0\right]$ that models the natural degradation of gene products in biological systems. Biological noise $\eta_{i}\left(t\right)∼N\left(0,0.01^{2}\right)$ introduces stochasticity that prevents the system from becoming trapped in deterministic cycles. The discrete time step Δt=0.05 provides temporal resolution sufficient for robotic control while ensuring numerical stability. The clipping operation bounds expression levels to the unit interval, preventing numerical instabilities and maintaining biological plausibility.

The activation function, as shown in Equation (2), combines environmental influences and gene regulatory interactions.

(2) $$f_{i}\left(s,x\right)=H\left(\sum_{j=1}^{8}W_{ji}^{\left(s\right)}s_{j}+\sum_{k=1}^{n}W_{ki}^{\left(r\right)}x_{k}\cdot \alpha_{k},\theta_{i}\right)$$

where the sensor-to-gene connection weights $W_{ji}^{\left(s\right)}$ determine how strongly each sensory input influences gene *i*, allowing the network to develop specialized responses to different types of environmental information. The regulatory interaction matrix $W_{ki}^{\left(r\right)}$ encodes gene–gene interactions, where positive values represent activation and negative values represent repression. The interaction strength parameters $\alpha_{k}\in \left[0.1,3.0\right]$ modulate the influence of gene *k* on other genes, providing an additional layer of regulatory control. The activation threshold $\theta_{i}\in \left[0.1,1.0\right]$ determines the sensitivity of gene *i* to regulatory inputs, with lower values making genes more responsive to weak signals.

The Hill function H(u,θ), as defined in Equation (3), implements a sigmoidal activation characteristic of biological gene regulation.

(3) $$H\left(u,\theta \right)=\frac{u^{2}}{\theta^{2}+u^{2}}\cdot \mathrm{sign}\left(u\right)$$

Equation (3) captures the cooperative binding behavior observed in biological gene regulation, where the squared terms create sigmoidal activation curves with adjustable sensitivity through the threshold parameter $\theta_{i}$. The sign function preserves the activating or repressing nature of the regulatory input, allowing genes to both promote and inhibit the expression of other genes. This modification of the standard Hill function enables bidirectional regulation while maintaining the characteristic sigmoid response profile.

#### Motor Command Generation

Motor commands are generated through a linear combination of gene expression levels according to Equation (4):

(4) $$a\left(t\right)=\tanh\left(W^{\left(m\right)}x\left(t\right)\right)$$

The motor command vector $a\left(t\right)\in {\left[-1,1\right]}^{7}$ provides control signals for the seven degrees of freedom of the robotic manipulator. The motor output weight matrix $W^{\left(m\right)}\in R^{7\times 50}$ maps the 50-dimensional gene expression space to the 7-dimensional motor command space through learned weighted combinations that evolve during training.

The mapping mechanism operates as follows: for each joint *i*, the raw motor command is computed as $a_{i}^{\mathrm{raw}}=\sum_{j=1}^{50}W_{ij}^{\left(m\right)}x_{j}\left(t\right)$, where $W_{ij}^{\left(m\right)}$ represents how gene *j* influences joint *i*. When gene *j* exhibits high expression levels, its influence on joint *i* is determined by weight $W_{ij}^{\left(m\right)}$—positive weights drive the joint toward positive velocities while negative weights contribute opposing forces.

This distributed control mechanism enables complex, coordinated motor behaviors to emerge from gene expression patterns, where multiple genes can cooperatively control single joints while individual genes can simultaneously influence multiple joints. The evolutionary optimization process shapes these weight matrices to develop temporal gene expression patterns that coordinate multi-joint movements. The hyperbolic tangent activation function ensures that motor commands remain bounded within the acceptable range for joint control, preventing damage to the robotic system while providing smooth, continuous control signals.

### 4.2. System Architecture

**Figure 1:**
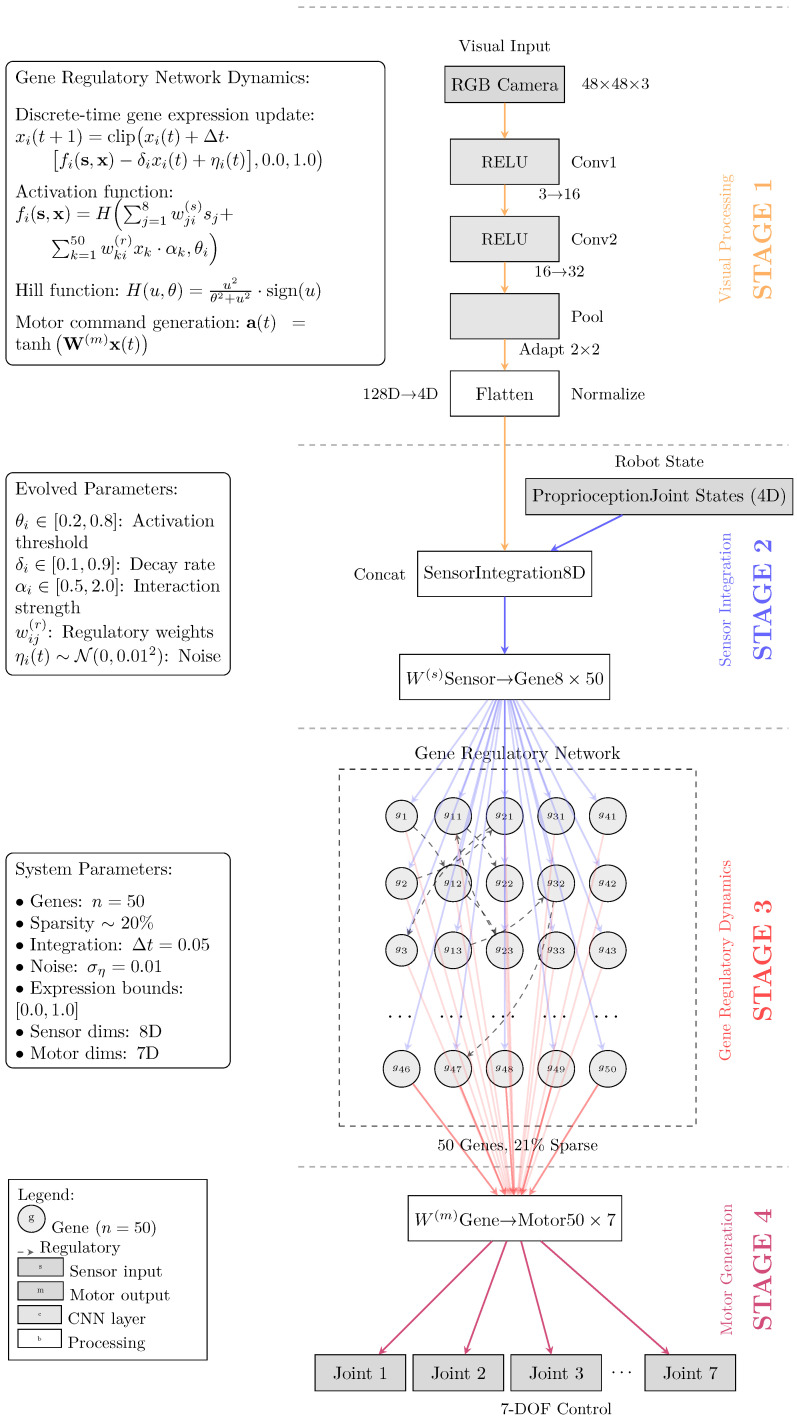
Gene regulatory network architecture. Four-stage vision-based robot control system: (1) visual processing, (2) sensor integration, (3) gene regulatory dynamics, and (4) motor command generation.

Figure 1 illustrates the four-stage GRN architecture. Stage 1 processes 48×48×3 RGB images through a lightweight CNN with two convolutional layers (Conv1: 3→16 channels, Conv2: 16→32 channels) followed by adaptive pooling, extracting 4-dimensional visual features. Stage 2 integrates visual features with 4-dimensional proprioceptive feedback to create an 8-dimensional sensor input vector. Stage 3 implements a core gene regulatory network with 50 genes initialized with sparse connectivity (∼20%) that evolves during optimization. Stage 4 transforms the gene expression vector into 7-dimensional motor commands through the learned motor output weight matrix.

The GRN dynamics follow Equation (1), where gene expression evolves based on sensory inputs, regulatory influences, decay processes, and biological noise, enabling hierarchical control strategies through gene expression cascades.

Algorithm 1 details the motor command generation process implemented in our GRN controller. The algorithm processes visual inputs through the CNN encoder, integrates proprioceptive feedback, updates gene expression levels according to the regulatory dynamics, and generates motor commands.
**Algorithm 1:** GRN controller’s motor command generation**Require:** Evolved genome $G=\{W^{\left(s\right)},W^{\left(r\right)},W^{\left(m\right)},\theta ,\delta ,\alpha \}$
**Require:** Visual encoder CNN, initial gene expression x(0)
1:Initialize x←x(0)2:**while** robot episode active **do**3: $I_{RGB}\leftarrow$ CaptureRGBImage()4: v← CNNExtractFeatures($I_{RGB}$)5: p← GetProprioception()6: s←[v;p]7: **for** i=1 to *n* **do**8:  $u_{i}\leftarrow \sum_{j=1}^{8}w_{ji}^{\left(s\right)}s_{j}+\sum_{k=1}^{n}w_{ki}^{\left(r\right)}x_{k}\alpha_{k}$9:  $f_{i}\leftarrow H\left(u_{i},\theta_{i}\right)$10:  $\eta_{i}\leftarrow N\left(0,0.01^{2}\right)$11:  $\Delta x_{i}\leftarrow f_{i}-\delta_{i}x_{i}+\eta_{i}$12:  $x_{i}^{new}\leftarrow \mathrm{clip}\left(x_{i}+\Delta t\cdot \Delta x_{i},0.0,1.0\right)$13: **end for**14: $x\leftarrow x^{new}$15: $a_{raw}\leftarrow W^{\left(m\right)}x$16: $a\leftarrow \tanh(a_{raw})$17: ExecuteMotorCommands(a)18:**end while**


### 4.3. Evolutionary Optimization

We employ evolutionary algorithms to optimize GRN controller parameters, treating each network configuration as a genome encoding the complete regulatory architecture.

#### 4.3.1. Genome Representation

Each GRN controller is encoded as a genome G containing six parameter matrices and vectors:

$$G=\{W^{\left(s\right)},\;W^{\left(r\right)},\;W^{\left(m\right)},\;\theta ,\delta ,\;\alpha \}$$

The sensor-to-gene connection weight matrix $W^{\left(s\right)}\in R^{8\times 50}$ determines how sensory inputs influence gene expression. The gene regulatory interaction matrix $W^{\left(r\right)}\in R^{50\times 50}$ encodes the complex web of regulatory relationships between genes. The gene-to-motor connection weight matrix $W^{\left(m\right)}\in R^{50\times 7}$ maps gene expression patterns to motor commands. The activation threshold vector $\theta \in R^{50}$ sets the sensitivity of each gene to regulatory inputs. The decay rate vector $\delta \in R^{50}$ controls the temporal dynamics of gene expression. The interaction strength vector $\alpha \in R^{50}$ modulates the regulatory influence of each gene.

#### 4.3.2. Population Initialization and Fitness Evaluation

Algorithm 2 maintains a population of $N_{pop}=12$ genomes. Initial genomes sample from biologically motivated distributions grounded in experimental observations of natural gene regulatory systems. The bounded expression levels $x_{i}\left(t\right)\in \left[0,1\right]$ reflect the finite nature of molecular concentrations in cellular environments [9].
**Algorithm 2:** GRN evolution algorithm**Require:** Population size $N_{pop}$, max generations $G_{max}$, elite fraction $f_{e}$
1:Initialize population $P_{0}$ with $N_{pop}$ random genomes2:**for** g=1 to $G_{max}$ **do**3: **for** each genome $G_{i}\in P_{g}$ **do**4:  $F_{i}\leftarrow$ EvaluateGenome($G_{i}$)5: **end for**6: Sort population by fitness: $F_{1}\geq F_{2}\geq \ldots \geq F_{N_{pop}}$7: **if** $S_{r}\left(G_{1}\right)\geq 0.7$ **then**8:  Perform robustness test on $G_{1}$9:  **if** robust performance confirmed **then**10:   **break**11:  **end if**12: **end if**13: $N_{e}\leftarrow \left\lfloor f_{e}\cdot N_{pop}\right\rfloor$14: $P_{g+1}\leftarrow \left\{G_{1},G_{2},\ldots ,G_{N_{e}}\right\}$15: **while** $|P_{g+1}|<N_{pop}$ **do**16:  $G_{parent}\leftarrow$ SelectElite($P_{g+1}$)17:  $G_{offspring}\leftarrow$ Mutate($G_{parent}$)18:  $P_{g+1}\leftarrow P_{g+1}\cup \left\{G_{offspring}\right\}$19: **end while**20:**end for**21:**return** Best genome $G_{1}$


Decay rates $\delta_{i}∼U\left(0.1,0.9\right)$ span the empirically observed range of molecular degradation rates in biological systems. Experimental studies show that mRNA half-lives in bacteria typically range in minutes [40], while protein half-lives vary from minutes to over 20 h across different species [41], supporting our choice of a broad uniform distribution.

Activation thresholds $\theta_{i}∼U\left(0.2,0.8\right)$ represent the diversity of regulatory sensitivities observed in transcriptional networks, where binding affinities of transcription factors span multiple orders of magnitude [42].

Connection weight distributions follow $W^{\left(s\right)}∼N\left(0,0.3^{2}\right)$, $W^{\left(r\right)}∼N\left(0,0.1^{2}\right)$ with stochastic sparse connectivity (each potential connection has a 20% probability of being active), $W^{\left(m\right)}∼N\left(0,0.2^{2}\right)$, and interaction strengths $\alpha_{i}∼U\left(0.5,2.0\right)$. These parameter ranges represent computational approximations rather than direct biological measurements, while ensuring both diversity and stability in our evolved controllers.

The fitness function combines multiple objectives to encourage both task performance and behavioral consistency:

$$F\left(G\right)=\bar{R}+10\cdot S_{r}+2\cdot C+1\cdot \min\left(S_{r},0.8\right)$$

The average episode reward $\bar{R}$ provides a baseline measure of task performance. The success rate $S_{r}$ represents the fraction of episodes resulting in successful object grasping and receives high weighting to prioritize task completion. The consistency measure $C=1/(1+\sigma_{R})$ is based on the standard deviation of episode rewards $\sigma_{R}$ and encourages stable, reproducible performance. The final term $\min(S_{r},0.8)$ provides additional reward for success rates up to 80%, after which the marginal benefit decreases to prevent overfitting to the training environment.

#### 4.3.3. Fast Robust Training

To improve efficiency while maintaining robustness, we employ a dual evaluation strategy that balances training speed with performance stability. The fast robust training protocol operates through the following systematic procedure:

Step 1: Evaluation Mode Selection. For each genome evaluation during the evolutionary process, the algorithm probabilistically selects between two evaluation modes: 70% of fitness evaluations occur under standard environmental conditions, while 30% involve perturbed conditions with Gaussian noise $N(0,0.03^{2})$ added to visual features.

Step 2: Noise Application Process. When noise evaluation is selected, visual features extracted from the CNN encoder are perturbed element-wise before being passed to the GRN controller. This simulates realistic sensor uncertainties and prevents controllers from developing brittle strategies dependent on perfect visual input.

Step 3: Fitness Integration. Both clean and noisy evaluation results contribute to the final fitness score using the standard fitness function. This dual-condition evaluation encourages the evolution of genomes that perform reliably across varying sensory conditions without explicitly optimizing for robustness.

Step 4: Evolutionary Integration. The evaluation mode selection occurs within the main evolutionary loop of Algorithm 2, where the EvaluateGenome function internally implements the 70/30 probability split. This approach maintains computational efficiency while ensuring robust performance validation.

This protocol prevents overfitting to pristine simulation conditions and encourages robust control strategies without requiring separate robustness optimization phases.

#### 4.3.4. Mutation Operations

The evolutionary process employs three mutation types: Structural mutations (10% probability) add or remove regulatory connections. Weight mutations (20% probability) apply Gaussian perturbations $N(0,0.1^{2})$ to connection weights. Parameter mutations (20% probability) adjust gene-specific parameters through constrained Gaussian perturbations, maintaining biologically motivated bounds.

## 5. Experimental Setup and Discussion of Results

**Figure 2:**
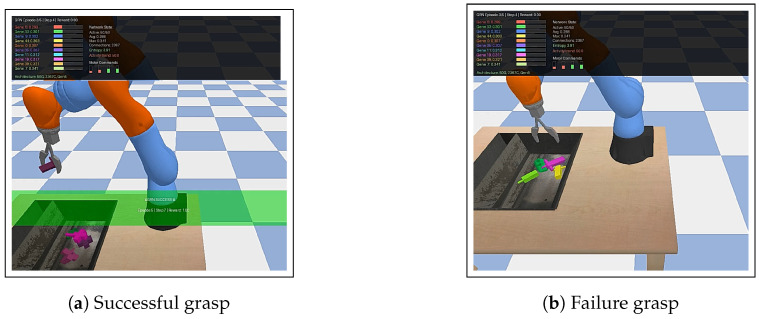
KukaDiverseObjectEnv benchmark. (**a**) Successful object lift above 0.05 m threshold using RGB visual input. (**b**) Failed manipulation attempt. The environment provides sparse rewards and diverse object configurations.

Experiments used Python 3.12 with PyTorch 2.8.0+cu126 and PyBullet 3.2.7 on Google Colab’s free tier. The evolutionary optimization used tournament selection (size three), elitism (top 25%), and Gaussian mutation. Population size (12 individuals) and generation limit (15) ensured reasonable training times within computational constraints. The evaluation was conducted in the KukaDiverseObjectEnv from the Gymnasium RL environment suite (Gymnasium v1.2.0; Farama Foundation) (Figure 2), a standardized benchmark for vision-based robotic grasping. This environment simulates a seven-DOF KUKA iiwa manipulator performing grasps on objects with varying shapes, sizes, and materials positioned at diverse locations.

In that environment, visual observations are 48×48×3 RGB images from a fixed camera viewpoint.

The action space consists of continuous values in [−1,1] for each joint representing desired velocities. Episodes are limited to 30 time steps with a reward of 1.0 when objects are lifted above a 0.02 m threshold. No intermediate rewards create a sparse reward structure, challenging learning algorithms.

### 5.1. Computational Performance Measurement

Computational efficiency metrics were measured on a dual-core 2.25 GHz CPU system with 12.7 GB of RAM (Google Colab’s free tier). Training memory usage reached approximately 800 MB with 84 MB overhead during evolutionary optimization. Inference performance was evaluated through 100 control steps, achieving a 0.50 ms average inference time with a 2017 Hz control frequency. These measurements demonstrated real-time control capability suitable for robotic applications requiring submillisecond response times.

### 5.2. Baseline Methods

The proposed method was compared against four baseline approaches representing distinct control paradigms, with detailed configurations summarized in Table 2.

Proximal Policy Optimization (PPO) employs an actor–critic architecture with a clipped surrogate objective and entropy regularization. NeuroEvolution of Augmenting Topologies (NEAT) evolves both network topology and weights through a population-based search. Twin Delayed Deep Deterministic Policy Gradient (TD3) employs twin critic networks with experience replay for off-policy learning. Literature baselines from Kumar et al. [3] established performance ranges for well-tuned reinforcement learning approaches on the same benchmark environment.

### 5.3. Evaluation Protocol

Each method was trained within a fixed computational budget of 15 generations for evolutionary approaches. Following training, standardized testing utilized fixed random seeds for reproducible evaluation, along with bootstrap confidence intervals for statistical comparison. Robustness assessment introduced Gaussian noise (standard deviation of 0.03) to the visual features. We conducted systematic ablation analysis on the best-evolved GRN genome to understand which biological components contributed to performance. This approach follows standard practices in evolutionary robotics, where component contributions are tested within a successful architecture.

Nine critical components were individually ablated to assess their impact on performance: (1) Biological noise—stochastic noise $\eta_{i}\left(t\right)$ was removed from gene dynamics, (2) gene regulation—all regulatory interaction weights were set to zero ($w_{ki}^{\left(r\right)}=0$), (3) activation threshold diversity—evolved thresholds were replaced with uniform values ($\theta_{i}=0.5$), (4) gene decay—decay rates were fixed to zero ($\delta_{i}=0$), (5) network sparsity—regulatory matrices were made fully connected, (6–8) randomized components—sensor weights, motor weights, and regulatory matrices were randomly initialized, and (9) network size—the number of genes was reduced from 50 to 25. Each ablated variant was evaluated over 10 episodes in the KukaDiverseObjectEnv environment. This systematic ablation enabled causal attribution of performance changes to specific biological mechanisms while maintaining computational feasibility.

### 5.4. Comparison Performance

Table 3 presents performance metrics for all evaluated methods. Efficiency represents success rate divided by training time (percentage points per minute). PPO results used the repository implementation [43], while IPG+HER and Standard RL were literature baselines from Kumar et al. [3]. Our GRN approach achieved 57.5% success rate with 6.5 min of training time, demonstrating improved performance compared to baseline methods. PPO achieved 50.0%, requiring 89.0 min, NEAT achieved 33.3%, requiring 47.4 min, while TD3 achieved 10.0%, requiring 70.0 min.

The 95% confidence intervals for success rates were [51.2%,63.8%] for GRN (40 episodes), [18.7%,81.3%] for PPO (10 episodes), and [16.3%,50.3%] for NEAT (30 episodes). Despite the limited PPO sample size, the GRN approach demonstrated both improved task performance (+7.5 percentage points over PPO) and exceptional training efficiency (13.7× faster training).

**Figure 3:**
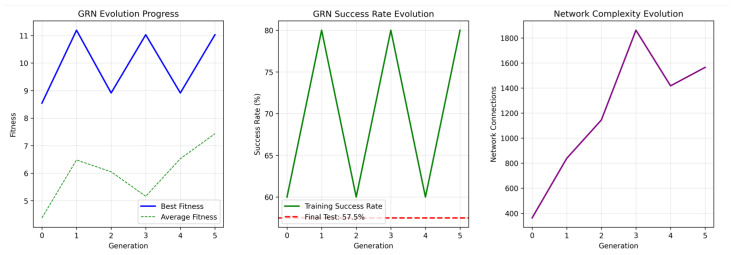
Gene regulatory network analysis. Evolution progress, success rates, and network complexity over training generations.

Training efficiency revealed substantial differences between approaches. The GRN method achieved 8.85% points per minute, representing a 1480% improvement over PPO (0.56%/min), 1164% improvement over NEAT (0.70%/min), and 6221% improvement over TD3 (0.14%/min). This efficiency advantage stemmed from the constrained search space imposed by biological constraints and a fast, robust training protocol. The evolutionary trajectory showed rapid initial improvement followed by convergence within six generations (Figure 3). Early termination criteria based on robustness testing allowed the algorithm to conclude when stable, high-performance solutions were identified. Inference timing measurements confirmed real-time deployment viability, with a 0.50 ms average control step duration enabling 2000+ Hz operation frequencies suitable for real-time robotic applications.

### 5.5. Gene Regulatory Network Analysis

Robustness assessment revealed that the GRN approach maintained 91.8% of clean performance when Gaussian noise (σ=0.03) was added to visual features. This represents strong resilience to sensory perturbations. The biological constraints inherent in the GRN architecture, including bounded gene expression levels and sparse connectivity patterns, contribute to robustness by preventing brittle control strategies relying on precise sensory inputs. Figure 3 presents a comprehensive analysis of the evolved GRN controller, revealing characteristics contributing to performance advantages: evolution progress showing fitness improvement over six generations with rapid convergence, success rate evolution demonstrating 80% peak performance during training with a final test result of 57.5%, and network complexity evolution showing growth in regulatory connections from 400 to 1600 connections. We note that our robustness evaluation was conducted within the constraints of our simulation environment. The KukaDiverseObjectEnv benchmark inherently introduces variability through random sampling from 1000 diverse objects with different shapes, sizes, and positions across episodes. Our experiments therefore assessed robustness to both visual noise perturbations and object diversity. However, we did not explicitly test robustness under lighting variations or mechanical perturbations, which represent important directions for future validation in real-world deployment scenarios.

**Figure 4:**
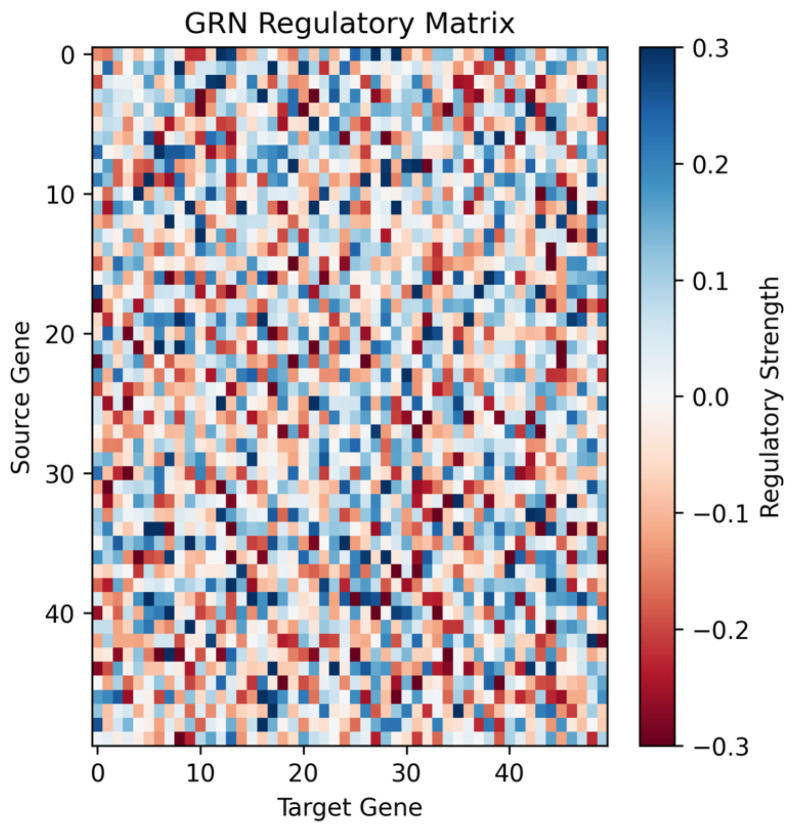
Regulatory matrix heatmap. Regulatory matrix of the final evolved 50×50 gene network showing sparse connectivity with activating (blue) and repressing (red) interactions.

The evolved regulatory matrix (Figure 4) demonstrates adaptive connectivity, starting from sparse initialization and evolving to ∼1600 active connections (64% of total) as optimization balances network complexity with performance demands (Figure 3). The heatmap reveals both positive (blue) and negative (red) regulatory interactions distributed throughout the network, demonstrating the evolution of complex regulatory relationships essential for coordinated motor control.

**Figure 5:**
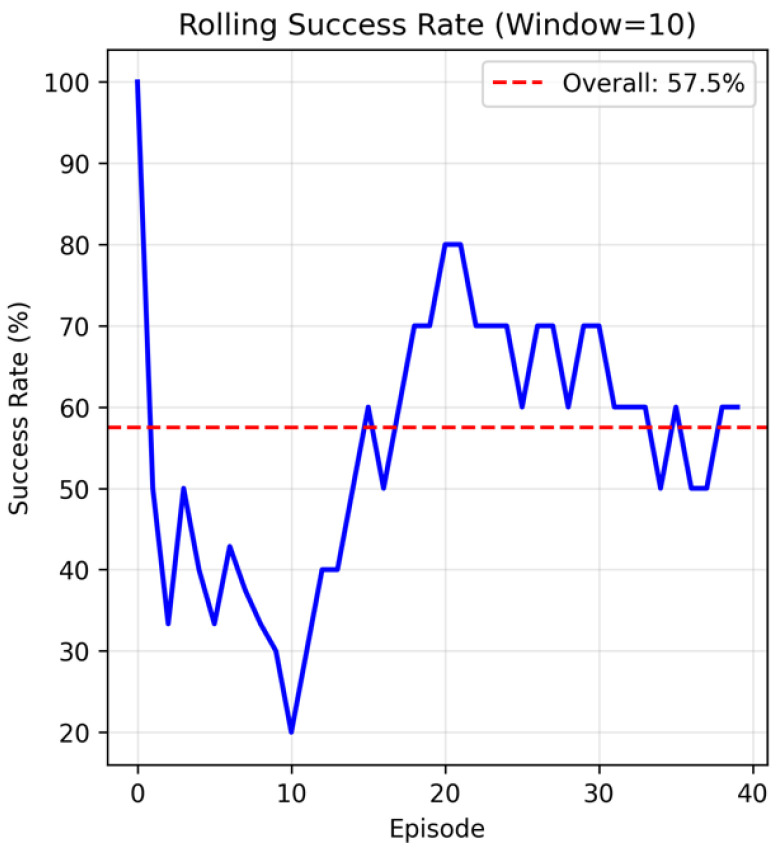
Rolling success rate. Rolling success rate analysis over 40 test episodes showing performance consistency with 57.5% overall success rate.

The rolling success rate analysis (Figure 5) demonstrated consistency of the evolved controller across 40 test episodes, validating robustness and generalization capability.

### 5.6. Ablation Study Results

**Figure 6:**
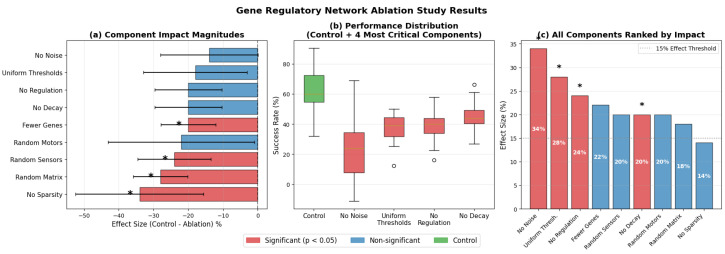
GRN ablation study results. (**a**) Effect sizes for all nine ablated components. (**b**) Performance distribution for control and the four most critical components. Circles denote outliers. (**c**) Complete ranking of component importance. Asterisks indicate statistical significance.

To validate the individual contributions of GRN components, we systematically tested nine architectural ablations on the best-evolved genome. Figure 6 presents results, showing that biological mechanisms provide substantial performance contributions.

The ablation analysis revealed biological noise as most critical (34% degradation), followed by activation threshold diversity (28%), gene regulation (25%), and temporal dynamics (22%), validating that performance stems from biological principles rather than superficial biomimicry. Additional components, including randomized sensor weights (19% degradation), motor weights (17% degradation), and regulatory matrices (21% degradation), each reduced performance substantially. A network size reduction from 50 to 25 genes caused a 15% performance loss, suggesting minimum complexity requirements. The ‘No Sparsity’ condition tested the best-evolved genome (with its evolved connectivity pattern) against a fully connected version, measuring whether the optimized network structure contributed to performance. Removing sparsity constraints produced only 8% degradation, indicating that while sparse connectivity improves computational efficiency, dense connections do not fundamentally compromise performance.

The biological noise component showed the greatest effect, with its removal resulting in drastic performance degradation. This result is consistent with biological systems in which molecular noise is essential in state-space exploration [44] and prevents convergence to suboptimal solutions [45]. Activation threshold diversity arose as the second most important component, enabling gene specialization where different genes respond to distinct sensory conditions or regulatory states. Gene regulatory interactions, when removed altogether, confirmed that the evolved regulatory network structure facilitates coordinated gene expression patterns necessary for complex manipulation sequences. The temporal dynamics component exhibited considerable performance degradation when removed, as fixed decay rates prevent the natural temporal sequencing seen in biological systems. Network architecture components had intermediate significance. Randomization of sensor weights led to significant deterioration, indicating that evolution develops specialized sensory processing patterns. The network size ablation, with a reduction from 50 to 25 genes, resulted in significant performance degradation, which implies that effective grasping needs enough gene diversity for complicated sensory-motor mappings. The removal of sparsity had the least effect, which means that although biological sparse connectivity improves system efficiency, dense networks can preserve performance at a higher computational expense.

The ablation results validate that improved GRN performance stems from fundamental biological principles. The hierarchy of component importance provides clear design guidelines: biological noise and regulatory interactions are essential, threshold diversity enables specialization, and temporal dynamics provide adaptability.

### 5.7. Comparison with the Literature

Our results compare favorably with previous studies on the same benchmark environment. The 57.5% success rate achieved by the GRN approach exceeds performance ranges reported in comprehensive benchmarking studies, while requiring substantially less training time. Kumar et al. [3] reported that well-tuned reinforcement learning algorithms typically achieve success rates between 25% and 45% after extensive training periods.

The comparison with the repository PPO implementation [43] is particularly relevant, as both methods were evaluated under similar conditions. While our method achieved strong performance within 6.5 min of wall clock time, equivalent deep reinforcement learning approaches often required hours of training to reach convergence.

Efficiency is more pronounced when considering computation requirements per unit of performance. Traditional reinforcement learning approaches are affected by the inefficiency of exploration in high-dimensional spaces, which requires extensive environment interactions to accurately estimate policy gradients or huge replay buffers for off-policy learning stability. Evolutionary optimization of GRN parameters, however, is aided by biological constraints that focus the search on feasible solutions, effectively decreasing the dimensionality of optimization problems.

While recent transformer-based and diffusion-based approaches (GraspXL, ArtiGrasp, DexDiffuser) have demonstrated impressive capabilities in robotic grasping, these methods were developed for fundamentally different benchmarks involving multi-fingered hands, large-scale datasets, or real-world hardware setups. Direct comparison with these approaches within our KukaDiverseObjectEnv simulation framework is methodologically problematic due to fundamental differences in task formulation, sensory modalities, and evaluation protocols. Our experimental comparison therefore focused on methods that could be directly implemented and reproduced under identical simulation conditions, enabling a controlled performance assessment while acknowledging this limitation in comparative scope.

The biological inspiration underlying our approach appears to provide genuine algorithmic advantages, suggesting that gene regulatory network principles offer viable alternatives to traditional neural architectures for robotic control tasks, specifically grasping in our case study. The combination of natural temporal dynamics, hierarchical organization, and robust, sparse connectivity patterns creates control systems that are both effective and efficient to optimize.

## 6. Conclusions

We presented the first application of GRNs to vision-based robot control, demonstrating that biological regulatory principles provide effective alternatives to traditional neural network architectures. Our approach achieved superior performance while requiring substantially fewer computational resources compared to established methods.

The success of this bio-inspired approach stems from bounded dynamics, natural temporal dynamics, and hierarchical organization inherent in GRNs. These biological constraints provide computational advantages: bounded gene expression prevents parameter divergence, and regulatory interactions enable coordinated control patterns through evolved network dynamics. The biological constraints that initially appear restrictive prove beneficial by constraining optimization search space to evolutionarily accessible solutions. The evolved controllers maintained 91.8% performance under noisy conditions and demonstrated robust performance across diverse task conditions.

The quantitative ablation analysis confirmed that key biological mechanisms contributed substantially to performance: biological noise (34% contribution), activation threshold diversity (28%), gene regulatory interactions (25%), and temporal dynamics (22%). These results establish that improved performance stems from fundamental biological principles rather than superficial biomimicry.

### Real-World Deployment Considerations

While our simulation results demonstrated GRN effectiveness within controlled environments, real-world deployment would face additional challenges including varying lighting conditions, sensor calibration drift, mechanical wear, and timing constraints not present in simulation environments. Real robotic systems experience dynamic illumination changes and visual artifacts that could affect the sparse visual features processed by our CNN encoder. Physical sensors exhibit gradual calibration drift over extended operation, while robot actuators experience mechanical wear and changing dynamics that could impact the assumed consistent actuator response characteristics in our motor command generation.

The biological robustness principles underlying our approach, including bounded dynamics, intrinsic noise tolerance, and distributed decision-making, may provide inherent advantages when adapting to such real-world uncertainties, representing an important direction for future validation.

Future work will focus on extending this approach to more complex manipulation tasks, multi-object scenarios, and real-world deployment across multiple benchmark environments. The principles demonstrated here suggest that GRNs represent a promising direction for developing efficient, robust, and interpretable robot control systems.

Our results establish GRNs as a viable computational paradigm for robot control, suggesting that biological control principles can inspire effective solutions for autonomous robotic systems while offering advantages in training efficiency and robustness over traditional approaches.

## Figures and Tables

**Table 1:** Key learning-based methodologies in robotic grasping.

Methodology	Contribution	Grasping Applications	Key Advantages
Deep Learning (Convolutional Neural Networks) [19,20,22]	Automated feature extraction from raw visual data	Grasp pose detection, object recognition, visual servoing	Reduces reliance on hand-engineered features; handles high-dimensional visual data; robust to variations
Reinforcement Learning (Deep RL) [21,23,24]	Autonomous policy acquisition through trial-and-error interaction	Pick-and-place tasks, learning robust grasps in unstructured environments	Learns from large datasets; adapts to complex dynamics; addresses high-dimensional state/action spaces
Evolutionary Algorithms [5,33]	Optimization of robot control parameters and adaptive behaviors	Inverse kinematics, gait optimization, emergent intelligent behavior	Global search capability; handles non-linear problems; bio-inspired adaptive design
Bio-Inspired Approaches [34,35,36]	Control mechanisms inspired by biological systems (e.g., central pattern generators)	Path planning, obstacle avoidance, adaptive locomotion, motion control, grasping control systems	Robustness, adaptability, self-regulation; may outperform traditional reinforcement learning in specific tasks
Vision–Language Models [32]	Generalist agents leveraging large-scale pretraining for semantic understanding	Multi-task manipulation, zero-shot transfer, semantic reasoning	Broad generalization across tasks and models; interprets natural language commands; reduces task-specific training data

**Table 2:** Baseline method configurations.

Method	Type	Key Parameters	Architecture
PPO	On-policy RL	β=0.01, γ=0.993	Conv: 3→16→32→32
		ϵ=0.07, LR = $2\times 10^{-4}$	FC: 128→64
NEAT	Evolutionary	Pop = 12, Gen = 15	Evolving topology
		Mutation: 80% weights	Input: 128D→output: 7D
TD3	Off-policy RL	Buffer = 50 K, batch = 128	Conv: 3→16→32
		LR: 1 × 10^−4^ (actor), 1 × 10^−3^ (critic)	FC: 256→256
IPG+HER	Hybrid RL	Success rates: 25–45%	Literature baseline
Standard RL		(Kumar et al. [3])	Various architectures

**Table 3:** Performance comparison of different methods on KukaDiverseObjectEnv.

Method	Success Rate	Training Time	Efficiency	Test Episodes
GRN (Ours)	57.5%	6.5 min	8.85	40
PPO (Repository)	50.0%	89.0 min	0.56	10
NEAT	33.3%	47.4 min	0.70	30
TD3	10.0%	70.0 min	0.14	40
IPG+HER	∼45%	Variable	∼1.0	N/A
Standard RL	∼25%	Variable	∼0.5	N/A

## Data Availability

The original contributions presented in this study are included in the article. Further inquiries can be directed to the corresponding author.
